# A newly recorded genus with description of a new cave-dwelling species of *Flagelliphantes* (Araneae, Linyphiidae) from northeastern China

**DOI:** 10.3897/BDJ.11.e105488

**Published:** 2023-05-29

**Authors:** Lan Yang, Zhiyuan Yao, Muhammad Irfan, Qiaoqiao He

**Affiliations:** 1 College of Life Science, Shenyang Normal University, Shenyang, China College of Life Science, Shenyang Normal University Shenyang China; 2 School of Life Sciences, Southwest University, Chongqing, China School of Life Sciences, Southwest University Chongqing China

**Keywords:** biodiversity, description, morphology, sheet-web spiders, taxonomy

## Abstract

**Background:**

The genus *Flagelliphantes* Saaristo & Tanasevitch, 1996 was proposed by Saaristo & Tanasevitch, 1996 to accommodate three *ex*-*Lepthyphantes* species distributed in northern Eurasia. Male *Flagelliphantes* are easlily recognised by having a hood-shaped thumb on the embolus. The females have a long, S-shaped scape and the posterior median plate of the epigyne is grossly enlarged (“hypertrophied”).

**New information:**

While examining Linyphiidae Blackwall, 1859 specimens from Yunxia Cave in China’s Jilin Province, we discovered a new cave-dwelling species of the genus *Flagelliphantes*, *F.yunxia* **sp. n.** In this paper, we provide detailed description and photos of its diagnostic somatic and genitalic features. It is the first record of the genus from China.

## Introduction

Linyphiidae is the second largest family of spiders, comprising 4,821 species in 635 genera distributed worldwide, including 64 fossil species and 22 genera (World Spider Catalog 2023). About 514 Linyphiidae species in 173 genera have been reported from China (Li 2020, Zhou et al. 2021, Irfan et al. 2021, Irfan et al. 2022a, Irfan et al. 2022b, Zhang et al. 2022, Irfan et al. 2023). The genus *Flagelliphantes* Saaristo & Tanasevitch, 1996 currently contains three species. *F.bergstromi* (Schenkel, 1931) is distributed in northern Europe, western, central and southern Siberia; *F.flagellifer* (Tanasevitch, 1988) is known from the Russian Far East; *F.sterneri* (Eskov & Marusik, 1994) is described from Sakhalin, Russia.

This paper adds another new species of this diverse family in China and constitutes the first record of genus *Flagelliphantes* of the country. It is a cave-dwelling spider, found in Yunxia Cave, located at Tonghua Municipality of Jilin Province in northeastern China, bordering the northern parts of the Democratic People’s Republic of Korea.

## Materials and methods

Specimens were examined and measured with a Leica M205 C stereomicroscope. Left male palp was photographed. Epigyne was photographed before dissection. Vulva was treated in a 10% warm solution of potassium hydroxide (KOH) to dissolve soft tissues before illustration. Images were captured with a Canon EOS 750D wide zoom digital camera (24.2 megapixels) mounted on the stereomicroscope mentioned above and assembled using Helicon Focus 3.10.3 image stacking software (Khmelik et al. 2005). All measurements are given in millimetres (mm). Leg measurements are shown as: total length (coxa, trochanter, femur, patella, tibia, metatarsus, tarsus). Leg segments were measured on their dorsal side. Metatarsal trichobothrium (Tm) is given as the ratio of the distance between the proximal margin of the metatarsus and the root of trichobothrium divided by the total length of the metatarsus (Denis 1949, Locket and Millidge 1953) and Tm value for the first and the fourth leg is given as TmI, TmIV, respectively. The specimens studied are preserved in 75% ethanol and deposited in the College of Life Science, Shenyang Normal University (SYNU) in Liaoning, P.R. China. Terminology and taxonomic descriptions follow Saaristo and Tanasevitch (1996).

**Abbreviations**

**Somatic morphology**: ALE = anterior lateral eye; AME = anterior median eye; AME–ALE = the distance between AME and ALE; AME–AME = the distance between AMEs; PLE = posterior lateral eye; PME = posterior median eye; PME–PLE = the distance between PME and PLE; PME–PME = the distance between PMEs.

**Male palp**: fg = Fickert’s gland; E = embolus; EP = embolus proper; LC = lamella characteristica; MM = median membrane; PC = paracymbium; PH = pit hook; R = radix; ST = subtegulum; T = tegulum; TA = terminal apophysis; TH = thumb.

**Epigyne**: EG = entrance groove; PMP = posterior median plate; PS = proscape; S = spermatheca; St = stretcher.

## Taxon treatments

### Flagelliphantes yunxia

Yao & Irfan sp. n.

F6637D05-23CC-562C-AB9D-549A7793A578

DF614288-D1A9-4972-A948-1DAFE0BECA10

#### Materials

- **Type status:** Holotype. **Occurrence:** recordedBy: Qiaoqiao He, Zhiyuan Yao; individualCount: 1; sex: male; lifeStage: adult; occurrenceID: 1F1EDAA3-0991-5FE5-84F8-16B052653512; **Taxon:** order: Araneae; family: Linyphiidae; genus: Flagelliphantes; **Location:** country: China; stateProvince: Jilin; municipality: Tonghua; locality: Erdaojiang District; verbatimLocality: Yayuan Town, Yunxia Cave; verbatimElevation: 461 m a.s.l.; verbatimLatitude: 41°45.850'N; verbatimLongitude: 126°12.633'E; **Event:** samplingProtocol: Collected by hand; year: 2019; month: 8; day: 2; **Record Level:** institutionCode: SYNU-Ar00297
- **Type status:** Paratype. **Occurrence:** recordedBy: Qiaoqiao He, Zhiyuan Yao; individualCount: 3; sex: 1 male, 2 females; lifeStage: adult; occurrenceID: B543A524-D3E4-51EC-81C3-EF0D21C710EC; **Taxon:** order: Araneae; family: Linyphiidae; genus: Flagelliphantes; **Location:** country: China; stateProvince: Jilin; municipality: Tonghua; locality: Erdaojiang District; verbatimLocality: Yayuan Town, Yunxia Cave; verbatimElevation: 461 m a.s.l.; verbatimLatitude: 41°45.850'N; verbatimLongitude: 126°12.633'E; **Event:** samplingProtocol: Collected by hand; year: 2019; month: 8; day: 2; **Record Level:** institutionCode: SYNU-Ar00298–00300

#### Description

**Male** (Holotype). Total length: 1.84. Carapace 0.85 long, 0.76 wide, yellow. Abdomen light yellow (Fig. 4A and B). Sternum 0.58 long, 0.63 wide. Clypeus 0.13 high. Chelicerae promargin with 2 teeth, retromargin with 4 teeth. Eye sizes and interdistances: AME 0.03, ALE 0.06, PME 0.05, PLE 0.06, AME-AME/AME 0.60, PME-PME/PME 0.80, AME-ALE/ALE 0.63, PME-PLE/PLE 0.86, coxae IV separated by 1.45 times their width. Length of legs: I 5.33 (0.26, 0.16, 1.30, 0.31, 1.28, 1.19, 0.83), II 4.60 (0.23, 0.13, 1.10, 0.29, 1.11, 1.02, 0.72), III 3.57 (0.20, 0.09, 0.84, 0.25, 0.80, 0.83, 0.56), IV 5.10 (0.24, 0.13, 1.27, 0.28, 1.25, 1.18, 0.75). Leg formula: I-IV-II-III. TmI 0.35, TmIV 0.25. Tibial spine formula: 2-2-2-2.

**Figure 1.**
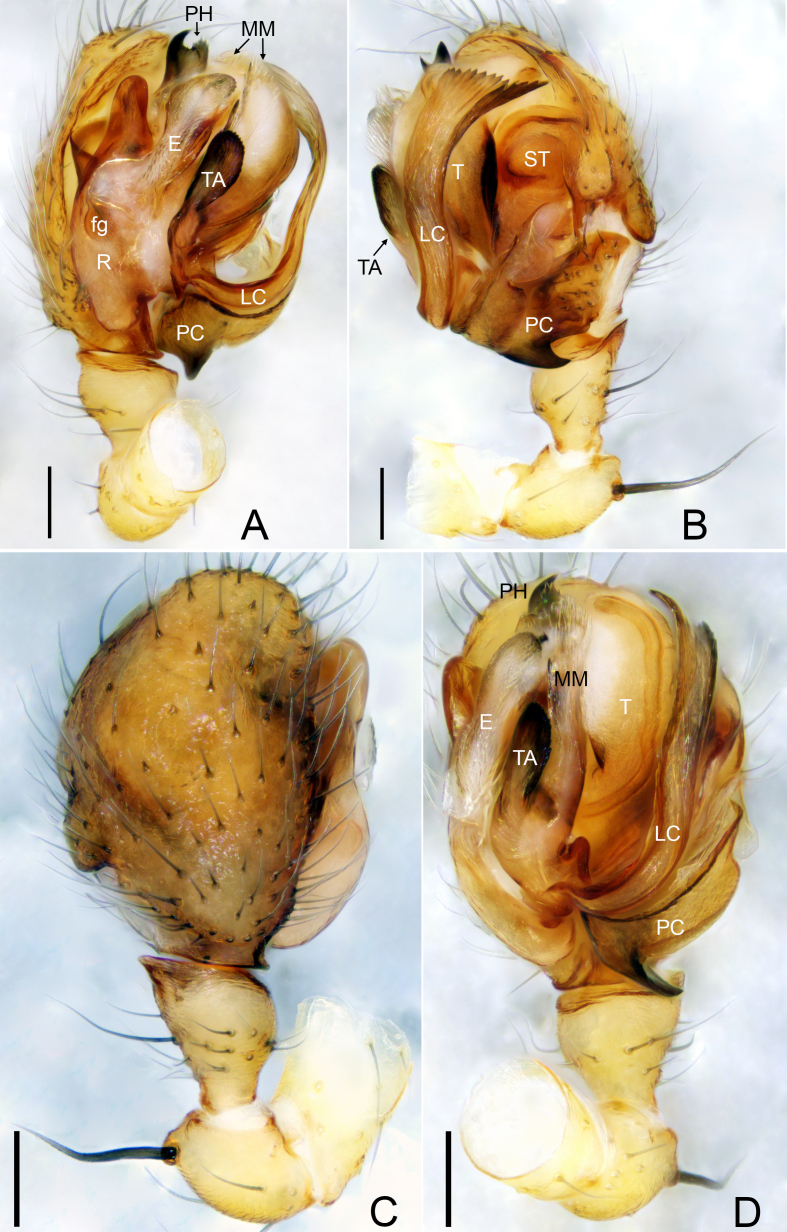
*Flagelliphantesyunxia* **sp. n.**, holotype male. **A** Palp, prolateral view; **B** Palp, retrolateral view; **C** Palp, dorsal view; **D** Palp, ventral view. Scale bars: 0.10 mm (A–D).

**Figure 2.**
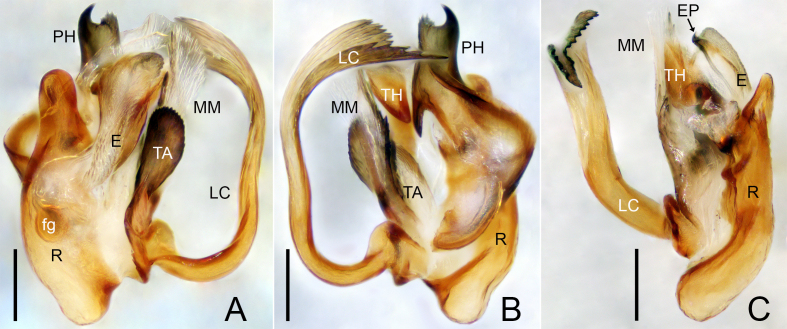
*Flagelliphantesyunxia* **sp. n.**, holotype male. **A** Embolic division, prolateral view; **B** Embolic division, retrolateral view; **C** Embolic division, lateral view. Scale bars: 0.10 mm (A–C).

Palp (Figs 1, 2). Patella almost as long as tibia, dorsally with thick macrosetae; tibia conical with two retrolateral trichobothria. Cymbium truncated; glabrous depression present at the base. Paracymbium basal part with microsetae, ventral margin with strongly sclerotised horn-shaped projection in retrolateral view, distal arm margin wave-like, with round tip. Pit hook longer than wide, mid dorsally with horn-shaped projection, pointing towards proximal part of lamella characteristica, apical part hook-shaped with seven teeth. Radix longer than wide, embolus elongated, slightly curved, embolus proper small; thumb hood-shaped. Lamella characteristica ribbon-shaped, apically abruptly broadened with 17 teeth.

**Female**. Total length: 2.23. Carapace 0.88 long, 0.73 wide, orange. Abdomen light yellow (Fig. 4C and D). Sternum 0.60 long, 0.54 wide. Clypeus 0.12 high. Chelicerae promargin with 3 teeth, retromargin with 3 teeth. Eye sizes and interdistances: AME 0.03, ALE 0.08, PME 0.06, PLE 0.07, AME-AME/AME 0.83, PME-PME/PME 0.89, AME-ALE/ALE 0.43, PME-PLE/PLE 0.54, coxae IV separated by 1.35 times their width. Length of legs: I 5.19 (0.26, 0.15, 1.30, 0.28, 1.25, 1.16, 0.79), II 4.61 (0.24, 0.13, 1.18, 0.24, 1.17, 1.06, 0.59), III 3.74 (0.20, 0.10, 0.96, 0.21, 0.89, 0.82, 0.56), IV 4.89 (0.24, 0.14, 1.20, 0.26, 1.21, 1.14, 0.70). Leg formula: I-IV-II-III. TmI 0.39, TmIV 0.15. Spine formula like in male.

**Figure 3.**
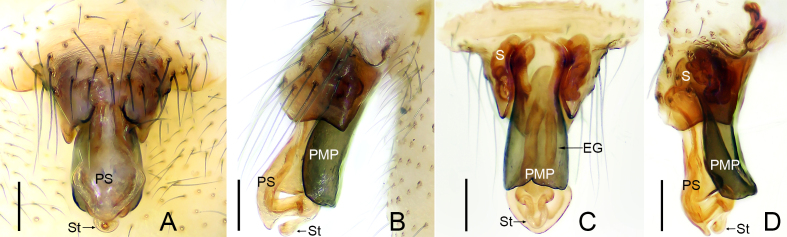
*Flagelliphantesyunxia* **sp. n.**, paratype female. **A** Epigyne, ventral view; **B** Epigyne, lateral view; **C** Vulva, dorsal view; **D** Vulva, lateral view. Scale bars: 0.10 mm (A–D).

**Figure 4.**
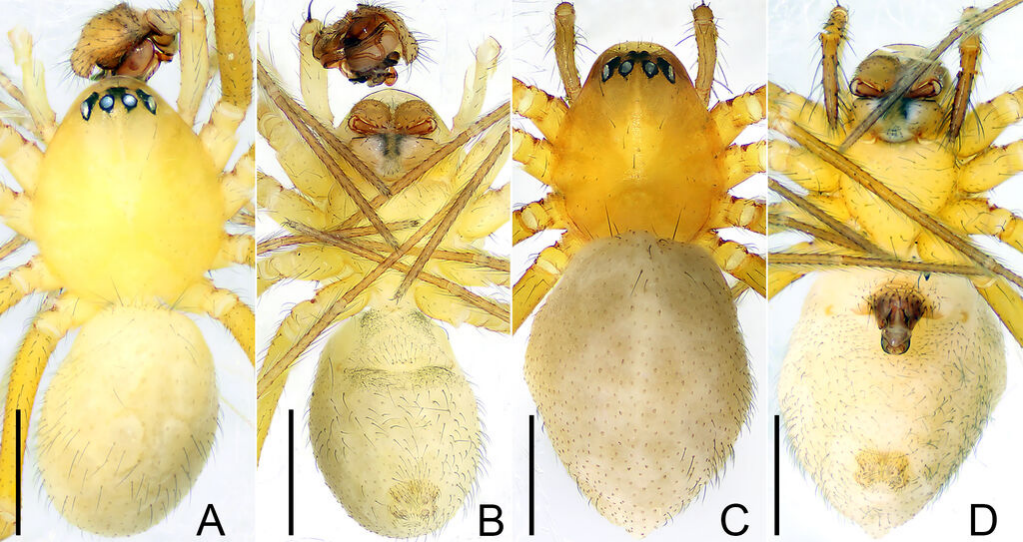
*Flagelliphantesyunxia* **sp. n.**, holotype male (A, B), paratype female (C, D). **A, C** Habitus, dorsal view; **B, D** Habitus, ventral view. Scale bars: 0.50 mm (A–D).

Epigyne (Fig. 3). Protruding; scape S-shaped, proscape long, slightly curved; both median and distal parts of scape short, lateral lobes poorly developed; stretcher distinct, relatively short; posterior median plate longer than wide, as long as proscape; spermathecae convoluted.

#### Diagnosis

The new species resembles *Flagelliphantesflagellifer* (Tanasevitch, 1988) described from the Kolyma Upland in northeastern Siberia by Tanasevitch (1988). In both cases, the male palps have similar ribbon-shaped “lamella characteristica”, both apically abruptly broadened with serrated margin; the epigynes are also similar with a broad, S-shaped scape (Figs 1, 2, 3; Tanasevitch (1988), figs 12, 14 and 15). The males of the two species can be distinguished by the following: apical part of terminal apophysis oval in the new species (Fig. 1A, Fig. 2A and B) vs. apical part elongated in *F.flagellifer* (Tanasevitch (1988), figs 12 and 13); apex of pit hook with seven teeth in the new species (Fig. 1A, Fig. 2A and B) vs. apex bifurcated in *F.flagellifer* (Tanasevitch (1988), fig. 12); lamella characteristica of *F.flagellifer* is much wider apically than in the new species (Fig. 1A, Fig. 2A and B; Tanasevitch 1988, fig. 13). The females can be distinguished by the following: posterior median plate as long as the proscape (Fig. 3) vs. half the length of proscape in *F.flagellifer* (Tanasevitch (1988), figs 14 and 15).

#### Etymology

The specific name refers to the type locality; noun in apposition.

#### Distribution

China (Jilin).

#### Biology

The species was found in the aphotic zone inside cave.

## Supplementary Material

XML Treatment for Flagelliphantes yunxia
